# Odevixibat after liver transplant in patients with progressive familial intrahepatic cholestasis type 1: A case series

**DOI:** 10.1002/jpn3.70227

**Published:** 2025-10-05

**Authors:** Georg‐Friedrich Vogel, Simone Kathemann, Andrea Pietrobattista, Giuseppe Maggiore, Denise Aldrian, Marco Sciveres, Henkjan J. Verkade, Etienne Sokal, Giulia Jannone, Magdalena Salcedo, Peter Rauschkolb, Christof Maucksch, Velichka Valcheva, Elke Lainka

**Affiliations:** ^1^ Department of Paediatrics I Medical University of Innsbruck Innsbruck Austria; ^2^ Institute of Cell Biology Medical University of Innsbruck Innsbruck Austria; ^3^ Department of Pediatric Gastroenterology Transplant Medicine, University Children's Hospital Essen Germany; ^4^ Hepatology and Liver Transplantation Unit, Bambino Gesù Children's Hospital IRCCS Rome Italy; ^5^ Pediatric Gastroenterology/Hepatology, Department of Pediatrics University of Groningen, Beatrix Children's Hospital/University Medical Center Groningen Groningen the Netherlands; ^6^ Université Catholique de Louvain, Cliniques St Luc Brussels Belgium; ^7^ Hepatology and Liver Transplantation Unit, CIBER‐Ehd Hospital General Universitario Gregorio Marañón Universidad Complutense Madrid Spain; ^8^ Ipsen Pharma GmBH Munich Germany; ^9^ Ipsen Ireland Dublin Ireland

**Keywords:** bile acids and salts, diarrhea, enterohepatic circulation, liver diseases, steatosis

## Abstract

**Objectives:**

Patients with progressive familial intrahepatic cholestasis type 1 (PFIC1) who have undergone liver transplantation (LT) may have unmet needs and impacts on daily life due to post‐LT complications, including diarrhea and hepatic steatosis. Here, we describe the effects of the ileal bile acid transporter inhibitor odevixibat on diarrhea and hepatic steatosis in a cohort of patients with PFIC1 post‐LT.

**Methods:**

Treating physicians from six centers retrospectively collected data through July 2023 on patients with PFIC1 who received odevixibat post‐LT. Data collected included demographics, medical history, and symptom presentation, characteristics of diarrhea, and liver imaging and/or histopathology.

**Results:**

Overall, nine male patients with PFIC1 (seven aged <18 years at initial completion of the case report form) were included. In most patients, the primary indication for odevixibat treatment was diarrhea and/or steatosis post‐LT. Odevixibat was initiated at a daily dose of 30−120 µg/kg (median exposure: 13 months). All patients had post‐LT diarrhea, which was generally associated with negative impacts on daily life (e.g., ability to attend school, needing to wear diapers due to fecal urgency). After odevixibat initiation, most patients had improved diarrhea and positive impacts on daily life. Among five patients with post‐LT steatosis and data available before and after odevixibat initiation, steatosis appeared to improve in three and did not change in two.

**Conclusions:**

Overall, the majority of patients with PFIC1 post‐LT complications in this case series experienced improvements in diarrhea and daily activities with odevixibat. Treatment with odevixibat following LT also appeared to reduce steatosis in some patients. Further studies, particularly those with a prospective design, are needed to confirm these findings.

## Abstract (highlights)

**What is Known:**

- Progressive familial intrahepatic cholestasis type 1 (PFIC1) is characterized by intractable pruritus, elevated serum bile acids, and progressive liver disease that may necessitate liver transplantation (LT).
- Diarrhea and steatosis are common complications in patients with PFIC1 following LT.
- Odevixibat is an ileal bile acid transporter inhibitor approved in the European Union for the treatment of PFIC and in the United States for the treatment of pruritus in PFIC.

**What is New:**

- Treatment with odevixibat in patients with PFIC1 post‐LT appeared to have clinical benefits, including improvements in diarrhea and improvements in aspects of daily life.
- Some patients also appeared to have improved steatosis with odevixibat.

## Graphical abstract

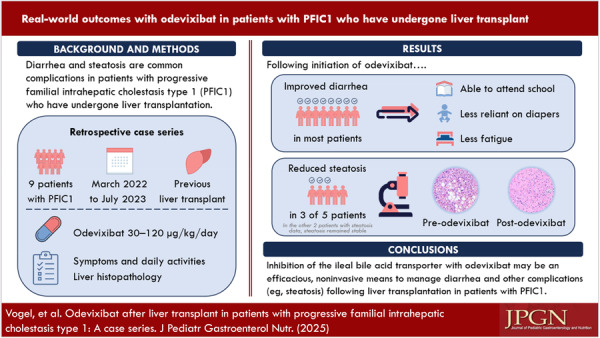

## 1 INTRODUCTION

The familial intrahepatic cholestasis 1 (FIC1) protein is an adenosine triphosphate‐dependent membrane transporter that is encoded by the *ATP8B1* gene and translocates phospholipids from the outer to the inner leaflet of the plasma membrane.^1^ FIC1 is expressed in multiple organs, including the liver, pancreas, and intestines.^1^, ^2^, ^3^ Mutations of *ATP8B1* can cause FIC1 deficiency, resulting in progressive FIC1 (PFIC1), which belongs to a broader family of PFIC diseases that, in total, are estimated to occur in approximately 1:50,000–1:100,000 births.^1^, ^4^ PFIC1 is characterized by both hepatic (e.g., cholestatic pruritus, elevated levels of serum bile acids, and progressive liver disease) and extrahepatic (e.g., impaired growth, malabsorption, diarrhea, pancreatic disease, and hearing loss) manifestations and frequently necessitates liver transplantation (LT) during childhood.^1^, ^5^

Potential complications in patients with PFIC1 who have undergone LT include hepatic steatosis, diarrhea with protein‐losing enteropathy, malabsorption, growth failure, pancreatitis, and poor graft survival.^6^, ^7^, ^8^, ^9^, ^10^, ^11^, ^12^, ^13^, ^14^, ^15^, ^16^ These outcomes, particularly diarrhea, steatosis, and poor graft survival, tend to be worse in patients with PFIC1 relative to other types of PFIC.^7^, ^9^, ^14^, ^15^ Exacerbations in diarrhea and other gastrointestinal symptoms post‐transplant may also impact quality of life (QoL) and contribute to the lack of catch‐up growth observed in many patients.^9^, ^12^, ^17^, ^18^

Studies to date have found that surgical biliary diversion (SBD) can be an efficacious means to alleviate diarrhea and prevent or reduce steatosis in some cases of patients with PFIC1 post‐LT.^13^, ^16^, ^19^, ^20^, ^21^, ^22^, ^23^ This procedure is designed to interrupt the enterohepatic circulation, and accordingly, reduce the size of the bile acid pool and decrease the rate of biliary bile acid secretion into the intestine.^24^ The invasive and complete nature of biliary diversion, however, necessitates hospitalization, introduces the risk of surgical complications, causes severe fat‐soluble vitamin deficiencies, and leads to postsurgery outcomes (e.g., an external stoma) that may be considered undesirable by patients and/or caregivers.^9^, ^13^, ^20^, ^25^ Accordingly, there is a need for a nonsurgical option to ameliorate diarrhea and preserve hepatic function in patients with PFIC1 who have undergone LT.

Inhibition of the ileal bile acid transporter (IBAT), also known as the apical sodium‐dependent bile acid transporter (ASBT), represents a pharmacological means of modifying the enterohepatic circulation of bile acids and a potential alternative to SBD.^11^, ^24^ IBAT inhibition was previously shown to lessen diarrhea in a patient with PFIC1 who underwent LT.^11^ Odevixibat is an orally administered, nonsystemic IBAT inhibitor that is currently approved for the treatment of PFIC in patients aged 6 months or older in the European Union and United Kingdom and for the treatment of pruritus in patients aged 3 months and older with PFIC in the United States.^26^, ^27^

In this case series, we describe the effects of odevixibat on diarrhea and steatosis in a cohort of patients with PFIC1 who have undergone LT.

## 2 METHODS

### 2.1 Ethics statement

In this case series, the families agreed to the evaluation of anonymized patient data in their treatment contracts, and each center also formally informed and signed a declaration of consent. Ethics committee approval for this study was granted from all treating institutions (i.e., Ethics Committee of the Medical University of Innsbruck; Ethics Committee of the Medical Faculty of the University of Duisburg‐Essen; Ethics Committee of the Bambino Gesù Children Hospital; Central Ethical Review Committee at the University Medical Center Groningen; Saint‐luc ‐ UCL Hospital‐Faculty Ethics Committee; Ethics Committee for Research With Medicines at the Gregorio Marañόn General Hospital).

### 2.2 Study design

Treating physicians from six sites in Europe retrospectively collected patient data using a standardized case report form with fields for demographic, clinical, and treatment information. All patients had undergone LT and received odevixibat post‐LT. Seven patients had a confirmed genetic diagnosis of PFIC1 (patients 1−5, 7, and 9). Patients 6 and 8, who are siblings and older than the other patients in the case series, each underwent an LT over 20 years ago at a pediatric hospital. Genetic testing was not standard at the time these patients underwent LT, and a diagnosis of PFIC1 was determined based on clinical presentation. These two patients entered the care of their current physician as adults (circa 2019); however, very few details from their pediatric medical histories remain and were available to be shared with the current physician.

Data collection occurred from March 2022 through July 2023 (as available). Clinical information collected included medical history and symptom presentation before and after LT, as well as before and after odevixibat initiation. Treatment outcomes included the following: characteristics of diarrhea and impacts of post‐LT diarrhea on daily life and activities; imaging and/or histopathology of the liver; laboratory parameters (e.g., serum bile acids, total bilirubin, alanine aminotransferase [ALT], aspartate aminotransferase [AST], gamma‐glutamyl transferase [GGT], albumin, international normalized ratio [INR]), and growth parameters (height, weight). Liver biopsies were conducted according to institutional protocols and/or were triggered by biochemical abnormalities (in the cases of patients 5 and 7). Steatosis was assessed by biopsy and liver elastography for patients 1, 2, and 7 and by biopsy for patients 4 and 5. Assessment of impacts on daily life was based on physician report; no formal QoL assessments were conducted.

## 3 RESULTS

### 3.1 Patient demographics and medical history before odevixibat initiation

In total, nine male patients (seven patients aged <18 years at the time the case report form was completed) with PFIC1 and a history of LT were included (Table 1). All nine patients were diagnosed in childhood (median [IQR] age at diagnosis: 4^10^ months; 7 of 9 patients with a genetically confirmed diagnosis of PFIC1). Before LT, major disease symptoms included pruritus, cholestasis, elevated serum bile acids, jaundice, and/or failure to thrive, and patients commonly received vitamin supplementation, rifampicin, cholestyramine, and/or ursodeoxycholic acid (UDCA). Patients 2 and 9 underwent SBD before LT. Indications for LT included decreasing liver function, cholestasis with intractable pruritus, and failure to thrive (Table 1). Median (IQR) age at the time of LT was 3^2^ years. A second LT was performed in patients 4 and 5. For patient 4, the second LT occurred 24 h after the first as a result of hepatic artery thrombosis. For patient 5, the second LT occurred approximately 3.5 years after the first and was due to progressive deterioration of graft function, severe steatohepatitis, and fibrosis. In addition, patient 5 underwent a total internal biliary diversion at the same time as the second LT; following these procedures, the patient's diarrhea improved but persisted. Post‐LT complications included diarrhea (all patients, including in patient 5 after both LT procedures), steatosis (patients 1, 2, 3, 4, 5, and 7), renal dysfunction secondary to chronic dehydration (patients 4 and 6), protein‐losing enteropathy (patient 7), and pancreatitis (patient 7).

**Table 1:** Demographics, medical history, and treatment information of patients with PFIC1 post‐LT.

	Odevixibat used for management of post‐LT complications								Odevixibat used for prevention of post‐LT complications
	Patient 1	Patient 2	Patient 3	Patient 4	Patient 5	Patient 6	Patient 7	Patient 8	Patient 9
*ATP8B1* gene [protein] mutation	[p.K455N; p.K455N]	c.2097+2T > C; c.2097+2T > C	c.1214_1215 del;c.(2931 + 1_2932‐1)_(3400 + 1_3401‐1) del	c.3040 C > T	c.1631‐1 G > C; c.1631‐1 G > C	Variants unknown	[p.R952X; p.T717N]	Variants unknown	c.1799 G > A; c.1799 G > A
Age at symptom onset	11 mo	2 mo	2 mo	1 mo	2 mo	Historical data not available	1 y	Historical data not available	2 mo
Age at PFIC diagnosis	1 y	4 mo	7 mo	<2 mo	4 mo	Neonatal	1 y	Neonatal	7 y
Surgical procedures before LT	NA	Biliary diversion with button implantation	None	None	None	Historical data not available	None	Historical data not available	Partial external biliary diversion
Age at LT	2 y, 9 mo	2 y, 10 mo	5 y	2 y, 4 mo; 2 y, 4 mo^a^	1 y, 7 mo; 4 y, 11 mo^b^	3 y	2 y, 10 mo	5 y	16 y
Symptoms leading to LT	Refractory pruritus Cholestasis Dystrophy Feeding disorder	Electrolyte imbalance Dystrophy Pruritus Infections with hospitalization	Intractable pruritus Decreasing liver function	1st LT: ◦ Cholestasis ◦ Jaundice ◦ Failure to thrive ◦ Severe pruritus 2nd LT: ◦ Hepatic artery thrombosis	1st LT: ◦ Cholestasis ◦ Failure to thrive ◦ Severe pruritus 2nd LT: ◦ Acidosis ◦ Failure to thrive ◦ Progressive liver damage	Historical data not available	Pruritus Failure to thrive Jaundice	Historical data not available	Refractory cholestasis Jaundice Pruritus
Surgical procedures after LT and before odevixibat	Port implantation Suspected intestinal invagination not confirmed intraoperatively	None	Surgical biliary diversion due to post‐LT steatohepatitis Biliary anastomosis was re‐inserted into the terminal ileum	None	Total internal biliary diversion at the same time as the second LT	None	Balloon dilatation of left sus‐hepatic vein stenosis Multiple endoscopic procedures with sphincter‐otomy, pancreatic duct dilatation and stenting for recurrent pancreatitis	Sessile serrated adenoma with low‐grade dysplasia located in sigmoid colon requiring endoscopic resection	NA
Time from LT to onset of diarrhea^c^	3 mo	6 mo	9 mo	12 y, 2 mo	4 mo	NA	1 y, 1 mo	NA	NA
Time from LT to odevixibat initiation	2 y, 0.5 mo	4 y, 11 mo	7 y, 10 mo	14 y, 9 mo	5 y	21 y, 7 mo	10 y, 10 mo	23 y, 4 mo	4 d
Starting daily dose odevixibat	~30 µg/kg	~100 µg/kg	~40 µg/kg	40 µg/kg	40 µg/kg	40 µg/kg	~40 µg/kg	40 µg/kg	120 µg/kg
Current daily dose odevixibat	~20 µg/kg	~100 µg/kg	~80 µg/kg	40 µg/kg	Discontinued	120 µg/kg	~25 µg/kg	120 µg/kg	120 µg/kg
Age at case report form completion	6 y	9 y	13 y	17 y	10 y, 7 mo	25 y	14 y	29 y	16 y
Treatment duration	21 mo	22 mo	17 mo	13 mo	8 mo	8 mo	10 mo	8 mo	19 mo^d^

Abbreviations: d, day; LT, liver transplantation; mo, month; NA, not available; PEG, percutaneous endoscopic gastrostomy; PFIC1, progressive familial intrahepaticcholestasis type 1; y, years.

^a^ Patient received a second LT approximately 24 h after the first due to hepatic artery thrombosis.

^b^ Patient received a second LT due to 2 episodes of acute rejection and portal anastomosis stenosis.

^c^ Based on first report of diarrhea captured in case report forms.

^d^ Patient was previously treated with odevixibat for approximately 15 months before undergoing LT; odevixibat was restarted approximately 4 days after LT.

After LT, patients commonly received UDCA, sodium bicarbonate, and immunosuppressive medications (e.g., tacrolimus, cyclosporine, azathioprine, mycophenolic acid, and prednisone). Surgical procedures performed after LT included implantation procedures (i.e., port implantation, feeding tube implantation; patient 1), internal biliary diversion to the terminal ileum (patient 3), and endoscopic procedures (patients 1, 2, 7, and 8). In patient 3 who underwent biliary diversion after LT, there was no improvement in diarrhea after diversion.

### 3.2 Odevixibat treatment

Persistent diarrhea and/or steatosis were reported as the primary indication(s) for initiating odevixibat in most patients. Among patients 1–8, the median (IQR) time from the final LT to odevixibat initiation was 6^6^ years in those aged <18 years at the time the case report form was completed and 22^1^ years in those aged >18 years at the time the case reported form was completed (Table 1). Patient 9 received odevixibat for approximately 16 months before LT for prolonged episodic cholestasis with treatment‐resistant pruritus and symptoms that substantially impacted daily activities (i.e., fatigue and jaundice). After starting odevixibat treatment and before LT, pruritus severity in patient 9 was reduced from severe to moderate but did not completely resolve, and the patient remained icteric; no diarrhea was noted. Immediately following LT, the patient experienced a brief period of diarrhea and resumed odevixibat; however, the relative timing of diarrhea onset and resolution and restarting of odevixibat is difficult to resolve because they both occurred during the same time frame (within days of the LT). Odevixibat was resumed to prevent common post‐LT complications.

Starting daily doses of odevixibat ranged from approximately 30–120 µg/kg across the nine patients (Table 1). The daily dose for patient 1 was initiated at ~30 µg/kg and reduced to ~15 µg/kg after 5 months due to gastroenteritis; the dosage was returned to ~30 µg/kg and subsequently decreased and maintained at ~20 µg/kg. For patient 2, the daily dose was initiated at a starting dose of ~100 µg/kg and decreased as low as ~70 µg/kg; as the lower doses were associated with an increase in diarrhea, the final dose was maintained at 90–100 µg/kg (i.e., 1600 µg/day). The daily dose for patient 3 was increased from ~40 to 80 µg/kg. Patients 6 and 8 underwent a planned dose increase of odevixibat from 40 to 120 µg/kg/day after a few weeks of treatment. Patient 7 initiated odevixibat at ~40 µg/kg; as the patient gained weight, no dose adjustment was made as the evolution of the patient's diarrhea and steatosis remained favorable, such that the patient was receiving a dose of ~25 µg/kg at the end of data collection. No dose adjustments were reported for patients 4, 5, and 9.

At the time of final data collection, median (IQR) exposure to odevixibat (including pre‐LT exposure to odevixibat for patient 9) was 13^11^ months, and 8 of 9 patients remained on treatment (Table 1). Patient 5 discontinued odevixibat after 8 months due to unchanged metabolic acidosis, hypokalemia, steatosis, and progression of liver disease despite mild improvement in stool frequency.

### 3.3 Concomitant medications before and after odevixibat initiation

Table, Supplemental Digital Content 2 describes treatment modulation by UDCA, sodium bicarbonate, and immunosuppressive medications before and after odevixibat initiation. Patient 1 required sodium bicarbonate before but not after odevixibat initiation, and patient 4 was able to decrease the dose of sodium bicarbonate by ~50% after starting odevixibat.

### 3.4 Diarrhea and impacts on daily life before and after odevixibat initiation

**Figure 1:**
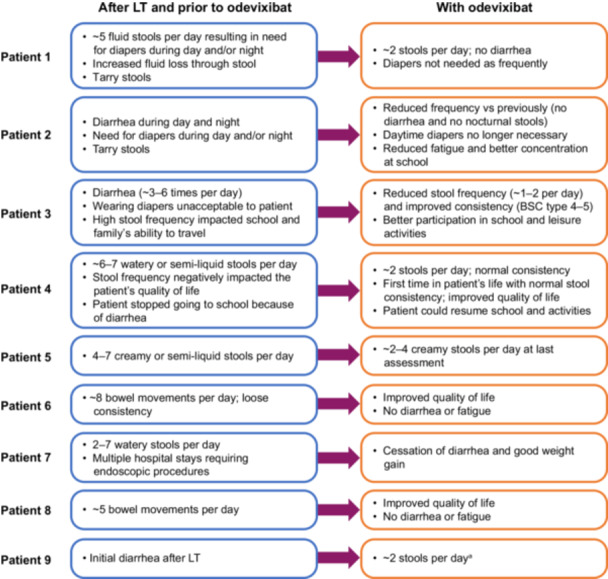
Changes in diarrhea and impacts on daily life in patients with PFIC1 post‐LT treated with odevixibat. ^a^Immediately following LT, the patient experienced a brief period of diarrhea and resumed odevixibat; however, the relative timing of diarrhea onset and resolution and restarting of odevixibat is difficult to resolve because they both occurred during the same time frame (within days of the LT). Odevixibat was resumed to prevent common post‐LT complications. Assessment of impacts on daily life was based on physician report. BSC, bristol stool chart, LT, liver transplantation; PFIC1, progressive familial intrahepatic cholestasis type 1.

Following LT, all nine (100%) patients reported diarrhea, and most reported negative impacts on daily life associated with post‐LT diarrhea (e.g., needing to wear diapers, interference with school attendance and family travel; Figure 1). Median (IQR) time from LT to onset of diarrhea was 1^1^ year in patients aged <18 years at the time of case report form completion (excluding patient 9, who received odevixibat before and immediately after LT; Table 1). In patients 6 and 8, due to paucity of data, the precise timing from LT to onset of diarrhea could not be determined; however, both patients were noted to have chronic diarrhea for years after LT, which impacted their growth, and in the case of patient 6, resulted in chronic renal disease due to diarrhea‐related dehydration. After odevixibat initiation, most patients had less frequent and more firm stools (Figure 1). Treating physicians noted that patients and their caregivers described improvements in multiple aspects of daily life, including less reliance on diapers and enhanced engagement in school and leisure activities.

### 3.5 Steatosis before and after odevixibat initiation

Median (IQR) time from LT to onset of steatosis was 10^5^ months (Table 2). Of the five patients with post‐LT steatosis and data available both before and after odevixibat initiation (patients 1, 2, 4, 5, and 7), 3 (60%) patients appeared to have improved steatosis with odevixibat based on histology (patients 1, 2, and 7 with treatment durations of 21, 22, and 10 months, respectively). In the remaining two (40%) patients, there was no apparent change in steatosis after odevixibat initiation (patients 4 and 5, with treatment durations of 13 and 8 months, respectively).

**Table 2:** Steatosis before and after odevixibat initiation in patients with PFIC1 post‐LT.

	Odevixibat used for management of post‐LT complications								Odevixibat used for prevention of post‐LT complications
	Patient 1	Patient 2	Patient 3	Patient 4	Patient 5	Patient 6	Patient 7	Patient 8	Patient 9
After LT and before odevixibat initiation									
Time to onset of steatosis	10 mo	11 mo	9 mo	~4 y	4 mo	NA	1 mo	NA	NA
Description of steatosis	10% macro‐vesicular and 5% micro‐vesicular fatty degeneration	25% macro‐ and 50% micro‐vesicular fatty degeneration	Post‐LT steatosis (80% of all hepatocytes) resolved with SBD 4 years post‐LT^a^	20%–35% micro‐/macro‐vesicular steatosis^b^	40% steatosis	No notable findings	40% steatosis (macro‐vesicular)	No notable findings	NA
With odevixibat^c^									
Description of steatosis	≤10% micro‐vesicular fatty degeneration	<5% micro‐vesicular fatty degeneration	NA	25%–30% micro‐/macro‐vesicular steatosis	40% steatosis	NA	No steatosis	NA	NA

Abbreviations: LT, liver transplantation; mo, month; NA, not available; PFIC1, progressive familial intrahepatic cholestasis type 1; SBD, surgical biliary diversion; y, year.

^a^ Odevixibat initiated due to persistent diarrhea.

^b^ Range from repeat biopsies before odevixibat initiation.

^c^ Data from last available biopsy.

Figures, Supplemental Digital Content 3, 4, and 5 show representative images of liver biopsies before and after odevixibat initiation for patients 1, 2, and 4, respectively.

### 3.6 Laboratory parameters before and after odevixibat initiation

**Figure 2:**
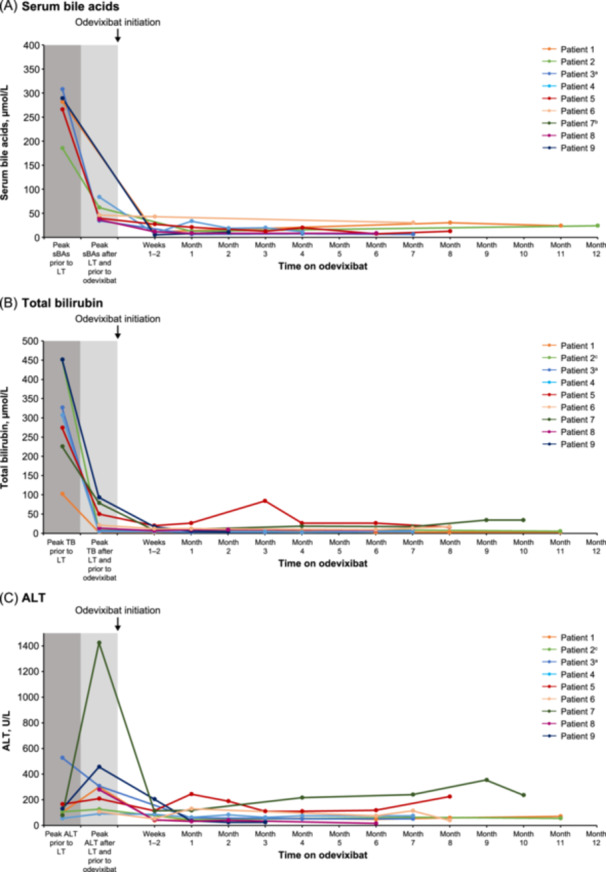
Serum bile acids (A), total bilirubin (B), and ALT (C) in patients with PFIC1 post‐LT treated with odevixibat. Values after odevixibat initiation are shown for the first 12 months of follow‐up. ^a^Patient data not shown for month 16 (bile acids: 7.8 µmol/L; total bilirubin: 5.1 µmol/L; ALT: 48 U/L). ^b^Only one bile acid value available for patient 7 (before LT: 121.1 µmol/L; not plotted). ^c^Patient data not shown for month 15 (total bilirubin: 3.4 µmol/L; ALT: 77 U/L). ALT, alanine aminotransferase; LT, liver transplantation; PFIC1, progressive familial intrahepatic cholestasis type 1; sBA, serum bile acid; TB, total bilirubin.

A total of six patients had serum bile acid values available after LT and before odevixibat initiation (Figure 2A). Relative to peak values post‐LT, odevixibat treatment resulted in lower serum bile acid levels that were sustained over time. During odevixibat treatment, four of nine patients had transient increases in total bilirubin levels that resolved with time (i.e., returned to or were lower than peak levels observed before odevixibat initiation). The remaining five patients had lower total bilirubin levels after odevixibat initiation compared with peak values before treatment (Figure 2B). Reductions in peak ALT were also observed after odevixibat initiation in seven of nine patients (Figure 2C). Of the four patients with albumin levels before and after odevixibat initiation, two showed increases in albumin with odevixibat to levels within the normal range (Table, Supplemental Digital Content 6). Changes in other liver‐specific parameters (i.e., AST, GGT, and INR) with odevixibat treatment are presented in Table, Supplemental Digital Content 6. Approximately half of patients had slight numeric increases in fat‐soluble vitamin levels (i.e., vitamins A, D, and E) after odevixibat initiation, and most patients had levels in the target range for each vitamin (Table, Supplemental Digital Content 7).

### 3.7 Growth parameters before and after odevixibat initiation

Height and weight increased over time in all patients with available data (Figure, Supplemental Digital Content 8) but generally remained below population norms throughout the course of odevixibat treatment. Representative graphs illustrating height and weight for patient 1 relative to standard growth percentiles are shown in Figure, Supplemental Digital Content 9.

### 3.8 Safety summary after odevixibat initiation

Two patients reported side effects with odevixibat (patients 7 and 8). Patient 7 had transient elevations in liver transaminase levels that resolved 2 weeks after the initiation of odevixibat. Patient 8 had a slight cutaneous eruption that occurred 7 days after starting odevixibat treatment; this event was nonspecific and self‐limiting. This patient also had transient increases in transaminase levels, which returned to normal levels after 10 days without intervention.

Patients 1 and 2 had a number of hospitalizations pre‐ and post‐LT due to gastroenteritis. Patient 1 was hospitalized for gastroenteritis six times before LT, 10 times post‐LT (but before odevixibat was started), and five times post‐LT and once odevixibat was started. Patient 2 was hospitalized for gastroenteritis four times before LT, 4 times post‐LT, and once post‐LT while receiving odevixibat. Patient 5 was hospitalized three times in total related to diarrhea; these instances occurred both before and after odevixibat initiation. Patient 7 was hospitalized twice due to diarrhea; the first instance occurred post‐LT as a result of gastroenteritis and the second as a result of protein‐losing enteropathy (odevixibat was initiated during the second hospitalization). Patients 3, 6, 8, and 9 were never hospitalized as a result of post‐LT diarrhea, and patient 4 had no hospitalizations related to diarrhea since starting odevixibat.

## 4 DISCUSSION

Diarrhea and steatosis are common complications in patients with PFIC1 who have undergone LT and may profoundly impact QoL and long‐term patient and graft survival.^7^, ^9^, ^10^, ^12^, ^17^, ^18^ Novel strategies to address these post‐LT complications are thus urgently needed. This case series of nine patients with PFIC1 who received odevixibat following LT suggests IBAT inhibition may represent a nonsurgical intervention to improve diarrhea and, in some patients, reduce steatosis.

All patients in the current series had post‐LT diarrhea. Previous research has suggested that this and other post‐LT complications result from the coupling of the native, FIC1‐deficient bowel and the physiological levels of bile acids secreted from the newly transplanted liver.^11^, ^19^ Specifically, a newly transplanted liver produces normal amounts of bile acid, and this high bile acid concentration relative to that before transplant is suspected to underlie post‐LT diarrhea.^6^, ^11^ By inhibiting IBAT, odevixibat diverts bile acids towards colonic excretion, and this is thought to reduce the overall size of the bile acid pool.^24^ Such a reduction could explain the effect of odevixibat on post‐LT diarrhea,^11^ although further research is needed to clarify these mechanisms.

Overall, eight of nine patients experienced improved diarrhea with odevixibat treatment (i.e., reduced frequency or improved consistency), and many reported improvements in aspects of daily life (e.g., engagement in school and leisure activities). Data from patients 2 and 3, who showed improvements in diarrhea with normalization of albumin levels during odevixibat treatment, also suggest the potential for resolution of protein‐losing enteropathy with odevixibat; however, given the small number of patients with these data available, additional study in this area is needed. Overall, the improvements in diarrhea and daily activities observed in this cohort are consistent with those previously reported in a patient with PFIC1 post‐LT who was treated with IBAT inhibitors (elobixibat followed by odevixibat).^11^

SBD has proven effective in alleviating diarrhea in some previous studies,^13^, ^16^, ^20^, ^21^, ^22^ although this does not always prevent or resolve diarrhea, likely due to an insufficient diversion approach and high concentrations of bile salts remaining in the large intestine,^28^ as well as ongoing malabsorption of essential fatty acids and fat‐soluble vitamins. This was the case for patient 3 in the current series who had an internal SBD to the terminal ileum performed post‐LT to prevent risk of ascending cholangitis.^20^ While this procedure did alleviate steatosis, diarrhea persisted following diversion. As the enterohepatic circulation of patient 3 was therefore still partly intact, additional pharmacological diversion by odevixibat was utilized to control the putatively chologenic diarrhea. Indeed, the patient experienced improvements in diarrhea consistency and frequency (from approximately 3−6 to 1−2 times per day) with odevixibat.

Steatosis develops in the majority of patients with PFIC1 experiencing post‐LT diarrhea and may be evident within the first month post‐transplantation.^7^, ^8^, ^10^, ^12^, ^21^, ^29^ In this series, six of nine patients had steatosis following LT, with time to onset ranging from 1 month to approximately 4 years. Liver changes with odevixibat treatment occurred on a spectrum, with three of five patients with available data appearing to show improved steatosis with odevixibat and two of five patients showing no notable change in steatosis. This difference in effectiveness across patients may reflect the underlying heterogeneity of the disease and warrants further study. It also remains possible that decreases in steatosis may be evident in additional patients (particularly those with improvements in liver‐specific laboratory values) as exposure to odevixibat increases.

Final dosing of odevixibat differed across patients and ranged from 30 to 120 µg/kg/day. Other than patient 2 (starting dose, 100 µg/kg/day) and patient 9 (who received odevixibat prophylactically), patients initiated odevixibat at 30–40 µg/kg/day and had their dose adjusted (or maintained) based primarily on either diarrhea response or a preplanned increase. In patient 2, diarrhea improved with initial dosing (100 µg/kg/day) but increased when the dose was lowered. The time from LT to odevixibat initiation varied widely and was directly related to the year in which odevixibat became available in the patient's geographic region compared with the year in which the patient underwent LT. The optimal time to initiate odevixibat treatment in patients with PFIC1 after LT remains unknown but is presumably at the time of symptom onset to impact both QoL and long‐term outcomes; however; use of odevixibat should be considered in light of its risk/benefit profile and any reimbursement considerations.

Limitations of the current study include the small sample size and the variability in dosing across 6 different centers. In addition, the retrospective nature of this case series is a limitation, although retrospective data collection is often necessary to build an evidence base in rare diseases like PFIC. Additional real‐world evidence such as data from registries could also be valuable. Also, data were limited to those collected during routine clinical management and were not uniformly available across patients; data were particularly limited for patients 6 and 8, who transferred from another medical institution before initiation of odevixibat. Finally, no formal assessments were used to assess impacts on daily life, and all included patients were male. Given these limitations, it is difficult to generalize the study findings, and results should be interpreted with caution. A prospective study that follows patients with PFIC1 who undergo LT and initiate odevixibat for post‐LT complications could help overcome the limitations inherent to a study with retrospective design and would be valuable for confirming these hypothesis‐generating observations.

## 5 CONCLUSIONS

Odevixibat treatment appeared to be associated with improvements in diarrhea and positive impacts on daily activities in most patients with PFIC1 post‐LT in this case series. Steatosis also appeared to improve in some patients. Additional studies, particularly those with a prospective design and larger sample size, are needed to confirm these findings.

## CONFLICT OF INTEREST STATEMENT

Georg‐Friedrich Vogel, has received scientific grant(s) and consultancy fees from Mirum, Takeda, and Ipsen. Andrea Pietrobattista has received consultancy fees from Mirum and scientific grant(s) from Ipsen. Giuseppe Maggiore has received consultancy fees from and has served as an investigator for Ipsen and Mirum; he has received consultancy fees from Alexion and Orphalan. Marco Sciveres has received consultancy fees from Ipsen and Mirum. Henkjan J. Verkade, has received consultancy fees from Ipsen, Intercept, Mirum, and Orphalan. Etienne Sokal has served as the Vice Chairman and Chairman of the Scientific and Medical Advisory Board for Cellaion; he has received consultancy fees from and has served as an investigator for Ipsen and has served as an investigator for Mirum and Intercept. Peter Rauschkolb, Christof Maucksch, and Velichka Valcheva were previously employed by Ipsen. Elke Lainka has received honoraria from Mirum. The remaining authors declare no conflict of interest.

## Supporting information


**LIST OF SUPPLEMENTAL DIGITAL CONTENT**

Plain Language Summary, Supplemental Digital Content 1.

**Table, Supplemental Digital Content 2.** Use of concomitant medications prior to and after odevixibat initiation in patients with PFIC1 post‐LT.

**Figure, Supplemental Digital Content 3.** Steatosis prior to (A) and after (B) odevixibat initiation in patient 1.

**Figure, Supplemental Digital Content 4.** Steatosis prior to (A) and after (B) odevixibat initiation in patient 2.

**Figure, Supplemental Digital Content 5.** Steatosis prior to (A) and after (B) odevixibat initiation in patient 4.

**Table, Supplemental Digital Content 6.** Laboratory values prior to and after odevixibat initiation in patients with PFIC1 post‐LT.

**Table, Supplemental Digital Content 7.** Fat‐soluble vitamin levels over time in patients with PFIC1 post‐LT.

**Figure, Supplemental Digital Content 8.** Height (A) and weight (B) prior to and after odevixibat initiation in patients with PFIC1 post‐LT.

**Figure, Supplemental Digital Content 9.** Height (A) and weight (B) of patient 1 relative to standard percentiles.
