# Supplementary material for: Odevixibat after liver transplant in patients with progressive familial intrahepatic cholestasis type 1: A case series
Source: J Pediatr Gastroenterol Nutr. 2025 Oct 5;81(6):1410–21. doi: 10.1002/jpn3.70227 (PMC12666498; doi:10.1002/jpn3.70227)
Supplement: Supplementary file 1 — Plain Language Summary, Supplemental Digital Content 1. [file JPN3-81-1410-s009.pdf]

Plain Language Summary, Supplemental Digital Content 1.

Patients with progressive familial intrahepatic cholestasis type 1 (PFIC1) have liver disease that worsens over time and may require liver transplantation (LT). Patients with PFIC1 who undergo LT may experience certain complications, including chronic diarrhea and a build-up of fat in the transplanted liver. These complications can affect quality of life and are sometimes treated using a surgical procedure called surgical biliary diversion.

Odevixibat is a medication that is taken by mouth and may help improve patient symptoms following LT without requiring additional surgery. This study describes 9 patients with PFIC1 who had severe diarrhea following LT and received odevixibat. Treatment with odevixibat led to improved diarrhea in most patients. In addition, most patients experienced improvements in their daily activities and were better able to participate in school and leisure activities.

Results from this study suggest odevixibat treatment could have important benefits in patients with PFIC1 who have undergone LT. Additional studies with more patients are needed to confirm these findings.
