# Supplementary material for: Odevixibat after liver transplant in patients with progressive familial intrahepatic cholestasis type 1: A case series
Source: J Pediatr Gastroenterol Nutr. 2025 Oct 5;81(6):1410–21. doi: 10.1002/jpn3.70227 (PMC12666498; doi:10.1002/jpn3.70227)
Supplement: Supplementary file 2 — Table, Supplemental Digital Content 2. Use of concomitant medications prior to and after odevixibat initiation in patients with PFIC1 post‐LT. [file JPN3-81-1410-s003.pdf]

Table, Supplemental Digital Content 2. Use of concomitant medications prior to and after odevixibat initiation in patients with PFIC1 post-LT

	**Odevixibat Used for Management of Post-LT Complications**								**Odevixibat Used for Prevention of Post-LT Complications**
**Time**	**Patient 1**	**Patient 2**	**Patient 3**	**Patient 4**	**Patient 5**	**Patient 6**	**Patient 7**	**Patient 8**	**Patient 9**
**Immediately prior to odevixibat initiation**	• Tacrolimus 0.8 mg BID<br>• UDCA 100 mg BID<br>• Sodium bicarbonate 8.4%, 10 mL TID	• Tacrolimus 0.8 mg BID<br>• Mycophenolic acid 180 mg BID<br>• UDCA 150 mg BID<br>• Sodium bicarbonate 8.4%, 15 mL BID<br>• Potassium chloride 600 mg BID<br>• Cholestyramine[a]	NA	• Tacrolimus 0.5 mg BID<br>• Sodium bicarbonate 8 g TID	• Cyclosporine 40 mg BID<br>• Azathioprine 12.5 mg/d<br>• Sodium bicarbonate 24 + 24 +24 + 30 mEq	• UDCA[a]	• Cholestyramine 4 g/d<br>• Sirolimus 3 mg/d	• UDCA[a]	• Prednisone 10 mg/d<br>• UDCA 600 mg/d
**Last available assessment after odevixibat initiation**	• Tacrolimus 1.5 mg BID<br>• UDCA 100 mg BID	• Tacrolimus 1 mg BID<br>• Mycophenolic acid 180 mg BID<br>• UDCA 200 mg BID<br>• Sodium bicarbonate 15 ml BID<br>• Potassium chloride 600 mg BID<br>• Omeprazole 10 mg BID	• Tacrolimus[a]	• Tacrolimus[a]<br>• Sodium bicarbonate 5 g TID	• Tacrolimus[a]<br>• Sodium bicarbonate 48+48+48 mEq<br>• Prednisone 5 mg EOD<br>• Azathioprine[a]	NA	• Sirolimus 3 mg/d<br>• Tacrolimus 5 mg/d	NA	NA

[a]Dosage not available. BID, twice a day; d, day; EOD, every other day; LT, liver transplantation; mEq, milliequivalent; NA, not available; PFIC1, progressive familial intrahepatic cholestasis type 1; TID, 3 times a day; UDCA, ursodeoxycholic acid.
