# Supplementary material for: Odevixibat after liver transplant in patients with progressive familial intrahepatic cholestasis type 1: A case series
Source: J Pediatr Gastroenterol Nutr. 2025 Oct 5;81(6):1410–21. doi: 10.1002/jpn3.70227 (PMC12666498; doi:10.1002/jpn3.70227)
Supplement: Supplementary file 3 — Figure, Supplemental Digital Content 3. Steatosis prior to (A) and after (B) odevixibat initiation in patient 1. [file JPN3-81-1410-s005.pdf]

Figure, Supplemental Digital Content 3. Steatosis prior to (A) and after (B) odevixibat initiation in patient 1

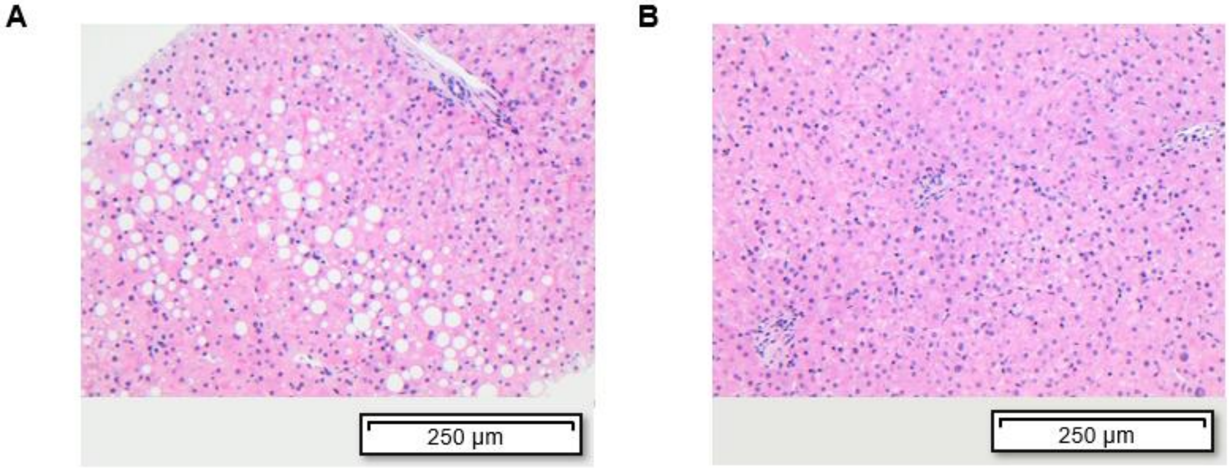

Panels A shows liver histology 10 months post-transplant; panel B shows liver histology 2 years post-transplant after 11 months of odevixibat treatment. A) Hematoxylin and eosin staining: Steatotic droplets of varying sizes (10% macrovesicular and 5% microvesicular fatty degeneration). B) Hematoxylin and eosin staining: Steatotic droplets of varying sizes (maximum 10% purely microvesicular fatty degeneration).
