# Supplementary material for: Odevixibat after liver transplant in patients with progressive familial intrahepatic cholestasis type 1: A case series
Source: J Pediatr Gastroenterol Nutr. 2025 Oct 5;81(6):1410–21. doi: 10.1002/jpn3.70227 (PMC12666498; doi:10.1002/jpn3.70227)
Supplement: Supplementary file 4 — Figure, Supplemental Digital Content 4. Steatosis prior to (A) and after (B) odevixibat initiation in patient 2. [file JPN3-81-1410-s008.pdf]

Figure, Supplemental Digital Content 4. Steatosis prior to (A) and after (B) odevixibat initiation in patient 2

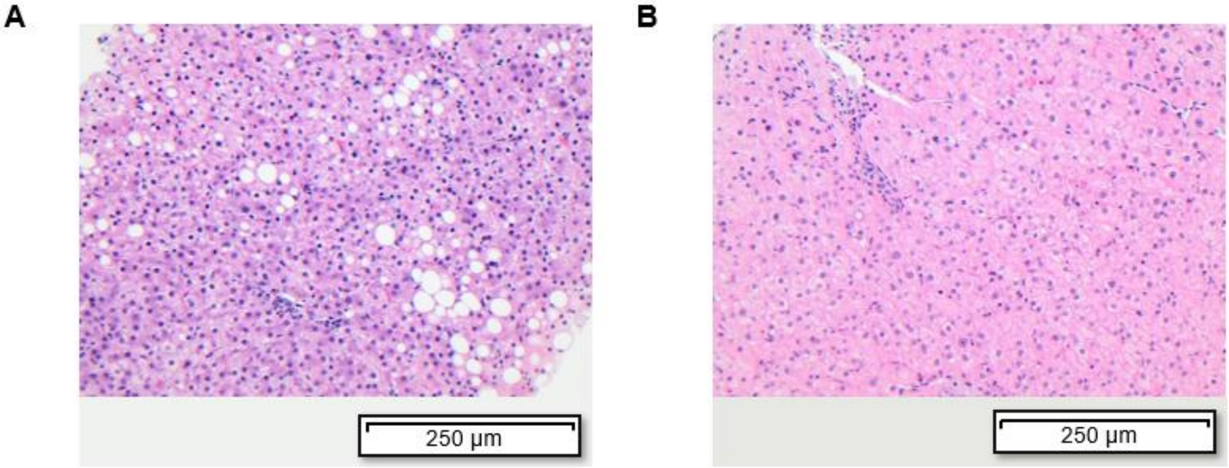

Panel A shows liver histology 11 months post-transplant; panel B shows liver histology 5 years post-transplant after 17 months of odevixibat treatment. A) Hematoxylin and eosin staining: Steatotic droplets of varying sizes (25% macrovesicular and 50% microvesicular fatty degeneration). B) Hematoxylin and eosin staining: Steatotic droplets of varying sizes (<5% microvesicular fatty degeneration).
