# Supplementary material for: Odevixibat after liver transplant in patients with progressive familial intrahepatic cholestasis type 1: A case series
Source: J Pediatr Gastroenterol Nutr. 2025 Oct 5;81(6):1410–21. doi: 10.1002/jpn3.70227 (PMC12666498; doi:10.1002/jpn3.70227)
Supplement: Supplementary file 5 — Figure, Supplemental Digital Content 5. Steatosis prior to (A) and after (B) odevixibat initiation in patient 4. [file JPN3-81-1410-s002.pdf]

**Figure, Supplemental Digital Content 5.** Steatosis prior to (A) and after (B) odevixibat initiation in patient 4

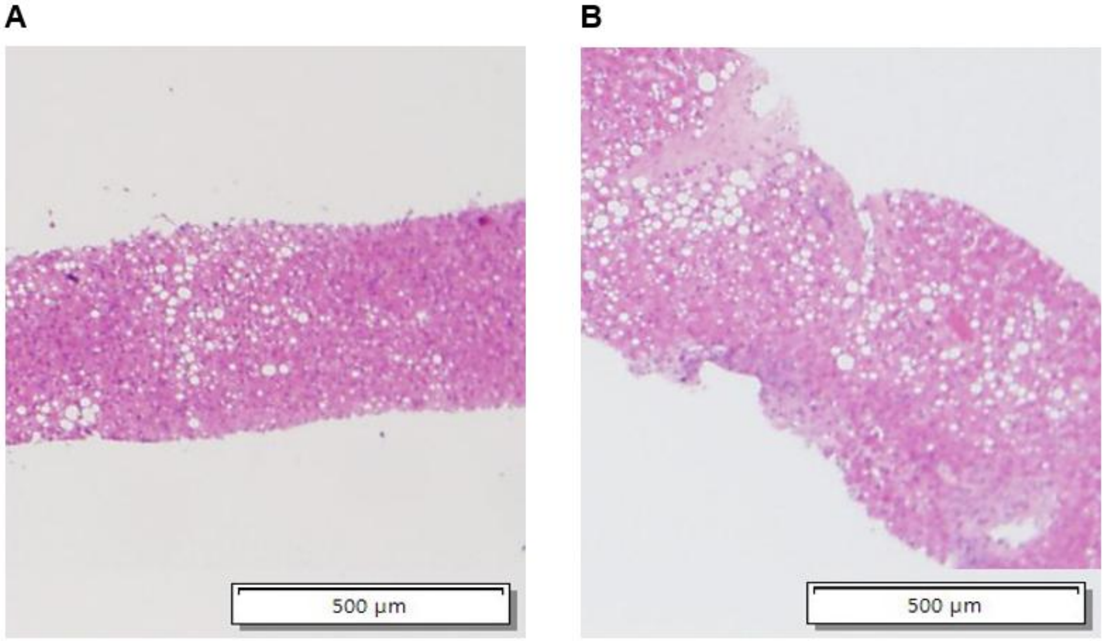


Panel A shows liver histology 10 years post-transplant; panel B shows liver histology 12 years post-transplant after 5 months of odevixibat treatment. A) Hematoxylin and eosin staining: Steatotic droplets of varying sizes (20% micro- and macrovesicular steatosis). B) Hematoxylin and eosin staining: Steatotic droplets of varying sizes (25−30% micro- and macrovesicular steatosis with mild portal and lobular inflammatory infiltrate).
