# Supplementary material for: Odevixibat after liver transplant in patients with progressive familial intrahepatic cholestasis type 1: A case series
Source: J Pediatr Gastroenterol Nutr. 2025 Oct 5;81(6):1410–21. doi: 10.1002/jpn3.70227 (PMC12666498; doi:10.1002/jpn3.70227)
Supplement: Supplementary file 6 — Table, Supplemental Digital Content 6. Laboratory values prior to and after odevixibat initiation in patients with PFIC1 post‐LT. [file JPN3-81-1410-s004.pdf]

Table, Supplemental Digital Content 6. Laboratory values prior to and after odevixibat initiation in patients with PFIC1 post-LT

	Patient 1	Patient 2	Patient 3	Patient 4	Patient 5[a]	Patient 6	Patient 7	Patient 8	Patient 9[b]
**AST, U/L**									
During follow-up after LT and prior to odevixibat initiation	99–165	61–103	87–321	43–60	33–223	28–64	22–1881	24–42	NA
During follow-up after odevixibat initiation	58–104	42–65	46–59	43–59	72–251	23–86	63–547	22–30	21–213
**GGT, U/L**									
During follow-up after LT and prior to odevixibat initiation	21	9–16	51–232	42–68	11–45	13–40	5–291	12–27	NA
During follow-up after odevixibat initiation	10–15	11–19	66–117	57–74	15–42	40–193	111–985	15–23	NA
**Albumin, g/dL**									
During follow-up after LT and prior to odevixibat initiation	3.4	2.5–3.7	2.7–3.3	4.0–4.5	NA	NA	2.5-4.8	NA	NA
During follow-up after odevixibat initiation	3.2–3.5	4.0–4.5	3.7–4.9	3.7–4.5	NA	NA	3.0-4.5	NA	NA
**INR**									
During follow-up after LT and prior to odevixibat initiation	1.1–1.2	1.1–1.3	1.2–1.5	1.1–1.4	1.3–2.2	1.1–1.4	1.1–1.5	1.1–1.2	NA
During follow-up after odevixibat initiation	1.0–1.2	0.9–1.0	1.3–1.4	1.2–1.4	1.3–1.9	1.0–1.1	1.1–1.4	NA	1.0–1.1

Values shown are ranges (as available). [a]Following second transplant and prior to odevixibat initiation. [b]Following odevixibat restart after LT. AST, aspartate aminotransferase; GGT, gamma-glutamyl transferase; INR, international normalized ratio; LT, liver transplantation; NA, not available; PFIC1, progressive familial intrahepatic cholestasis type 1.
