# Supplementary material for: Odevixibat after liver transplant in patients with progressive familial intrahepatic cholestasis type 1: A case series
Source: J Pediatr Gastroenterol Nutr. 2025 Oct 5;81(6):1410–21. doi: 10.1002/jpn3.70227 (PMC12666498; doi:10.1002/jpn3.70227)
Supplement: Supplementary file 7 — Table, Supplemental Digital Content 7. Fat‐soluble vitamin levels over time in patients with PFIC1 post‐LT. [file JPN3-81-1410-s007.pdf]

Table, Supplemental Digital Content 7. Fat-soluble vitamin levels over time in patients with PFIC1 post-LT

	**Vitamin A, μg/dL** Target range: > 20 μg/dL (1,2)			**Vitamin 25OH-D, ng/mL** Target range: 20−60 ng/mL (1,2)			**Vitamin E, mg/dL** Target range: >0.7 mg/dL (2)		
	Prior to LT	Post-LT	After odevixibat initiation	Prior to LT	Post-LT	After odevixibat initiation	Prior to LT	Post-LT	After odevixibat initiation
**Patient 1**	116[a]	28	21	9.8[a]	58[a]	47	4.7[a]	6.4	8.5
**Patient 2**	54[a]	29	23	22[a]	51	46	5.1[a]	5.5	6.4
**Patient 3[a]**	210	320	350	18	23	25	3.2	4.0	2.8
**Patient 4**	−	57	40	−	<14[a,b]	<4.4[a,b]	−	<0.4	<0.3[b]
**Patient 5[a]**	−	1	8.8−14	−	3.2	12−36	−	0.2	0.2−0.6
**Patient 6[a]**	−	1.6	14.6	−	45	35	−	0.04	0.12
**Patient 7**	47−101[a]	14−59[a]	47−62[a]	13−28[a]	4−37[a]	28−32[a]	0.8−1.0[a]	0.3−0.9	0.8−1.1
**Patient 8[a]**	−	3.0	6.3	−	26	46	−	0.08	0.07
**Patient 9[a]**	54	−	49−66	15	−	65	1.0	−	1.0−1.8

[a]Patient receiving vitamin supplementation. [b]Dose of vitamin supplementation raised based on these results. −, not available; LT, liver transplantation; PFIC1, progressive familial intrahepatic cholestasis type 1.

References

1 Kamath BM, Alonso EM, Heubi JE, et al. Fat soluble vitamin assessment and supplementation in cholestasis. *Clin Liver Dis* 2022;26:537-53.

2 Degrassi I, Leonardi I, Di Profio E, et al. Fat-soluble vitamins deficiency in pediatric cholestasis: A scoping review. *Nutrients* 2023;15.
