# Supplementary material for: Odevixibat after liver transplant in patients with progressive familial intrahepatic cholestasis type 1: A case series
Source: J Pediatr Gastroenterol Nutr. 2025 Oct 5;81(6):1410–21. doi: 10.1002/jpn3.70227 (PMC12666498; doi:10.1002/jpn3.70227)
Supplement: Supplementary file 8 — Figure, Supplemental Digital Content 8. Height (A) and weight (B) prior to and after odevixibat initiation in patients with PFIC1 post‐LT. [file JPN3-81-1410-s001.pdf]

Figure, Supplemental Digital Content 8. Height (A) and weight (B) prior to and after odevixibat initiation in patients with PFIC1 post-LT

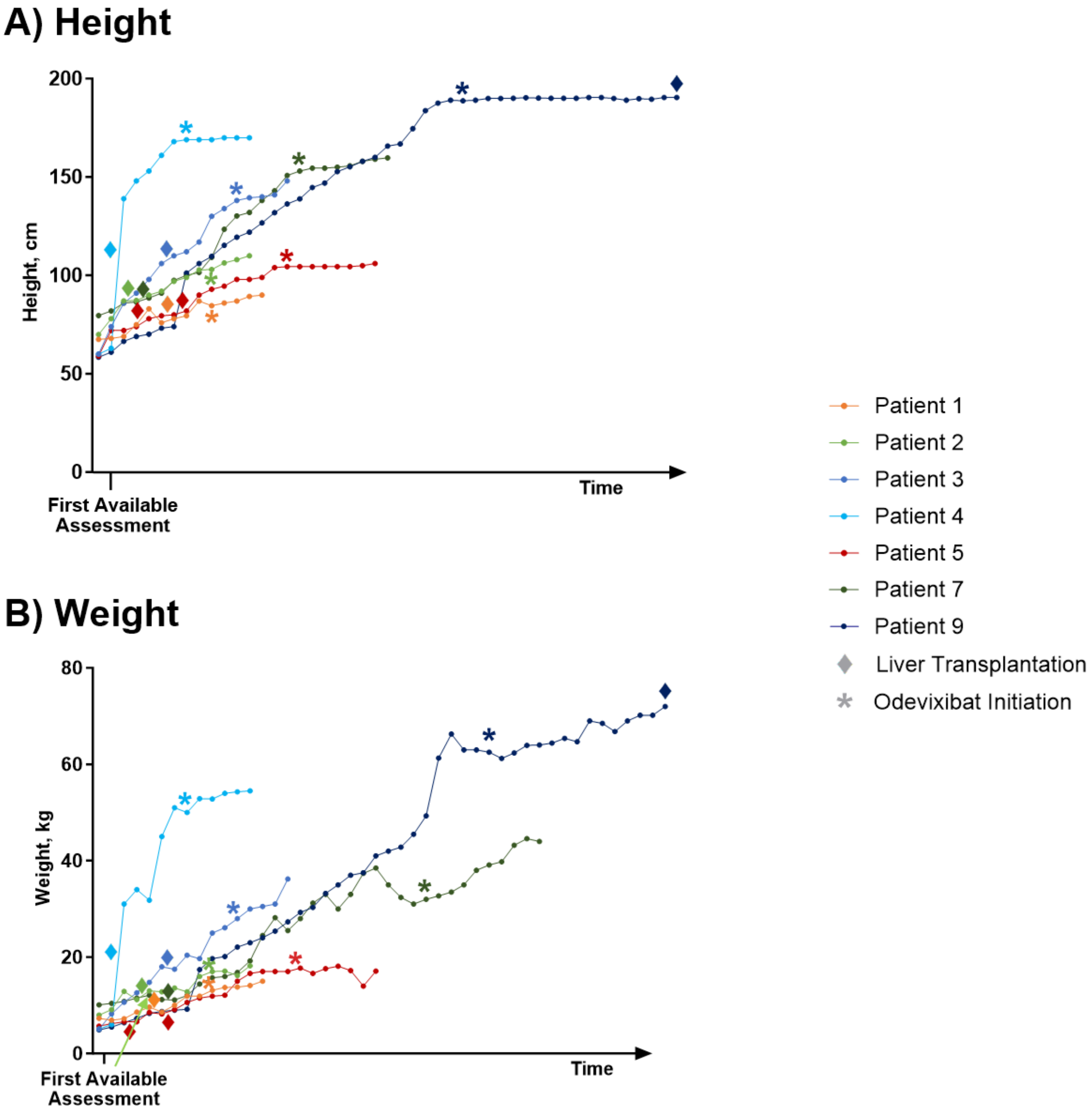

Patients 6 and 8 had only 1 growth assessment available and are not plotted here. For each of these 2 patients, assessment occurred 8 months after odevixibat was initiated. The height and weight values for patient 6 were 151 cm and 31 kg, respectively, and for patient 8 were 154 cm and 38 kg, respectively. LT, liver transplantation; PFIC1, progressive familial intrahepatic cholestasis type 1.
