# Supplementary material for: Odevixibat after liver transplant in patients with progressive familial intrahepatic cholestasis type 1: A case series
Source: J Pediatr Gastroenterol Nutr. 2025 Oct 5;81(6):1410–21. doi: 10.1002/jpn3.70227 (PMC12666498; doi:10.1002/jpn3.70227)
Supplement: Supplementary file 9 — Figure, Supplemental Digital Content 9. Height (A) and weight (B) of patient 1 relative to standard percentiles. [file JPN3-81-1410-s006.pdf]

Figure, Supplemental Digital Content 9. Height (A) and weight (B) of patient 1 relative to standard percentiles.

A) Height

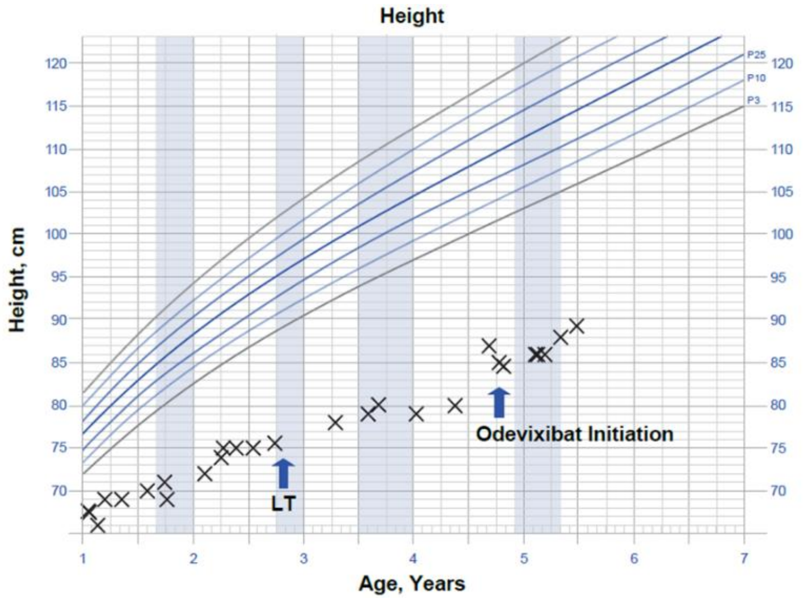

B) Weight

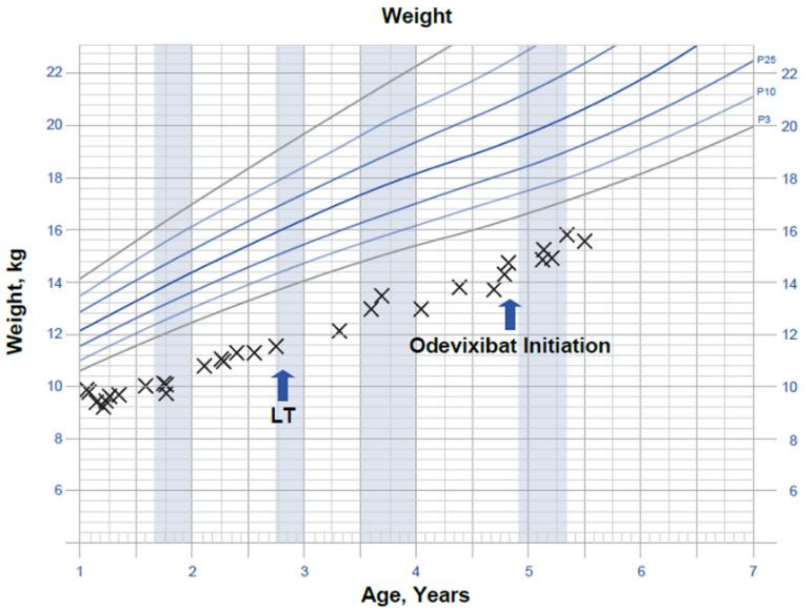

Height and weight increased in patient 1 following odevixibat initiation. Catch-up growth was minimal, as the patient remained below the 3rd percentile for both height and weight. LT, liver transplantation.
